# Managing stress granule disassembly with ubiquitin and its cousin

**DOI:** 10.1038/s41392-021-00782-2

**Published:** 2021-11-11

**Authors:** Stefan Müller

**Affiliations:** Institute of Biochemistry II, Gustav Embden Zentrum, Goethe University Frankfurt, Faculty of Medicine, Frankfurt, Germany

**Keywords:** Biochemistry, Neurology

Recently, Maxwell et al.^1^ revealed a critical function of ubiquitylation in stress granule (SG) disassembly upon recovery from heat stress.

Organisms are continuously exposed to endogenous or environmental stress. Genotoxic stress endangers DNA integrity, whereas proteotoxic stress causes an imbalance in protein homeostasis (proteostasis). Genotoxic insults activate DNA damage response pathways that halt the cell-cycle progression and initiate DNA repair. Protein quality control (PQC) systems in turn safeguard the integrity of the proteome and prepare for the restart of cellular activities upon stress release. Both genome and proteome integrity pathways are orchestrated by ubiquitin signaling and the ubiquitin-proteasome system (UPS). Here, we highlight very recent elegant recent work by the Taylor laboratory providing novel insight into how cells exploit the ubiquitin system for recovery from heat stress.^1,2^

Proteostasis is accomplished by a network of pathways that balance protein synthesis, folding, transport, and disposal. Proteotoxic stress disturbs the equilibrium of these processes and typically induces the misfolding of nascent and mature proteins, which can ultimately lead to their loss of function or trigger the formation of toxic protein aggregates. Neurodegenerative diseases are prime examples of protein misfolding diseases (aka proteinopathies), in which protein aggregates impair critical cellular functions and cause irreversible damage to cells, tissues, and organs. As one line of defense against protein misfolding, chaperone systems are activated. As a second line, misfolded proteins are cleared by the autophagosome/lysosome or the UPS. The inhibition of splicing, nucleocytoplasmic transport, and protein synthesis serves as an additional safeguard mechanism for maintaining protein homeostasis under stress by avoiding further influx into the overloaded proteostasis systems. The limitation of translation is tightly linked to the formation of distinct cytosolic ribonucleoprotein condensates, termed SGs. SG formation is triggered by the accumulation of ribosome-free mRNAs generated upon stalling of translation initiation. The transient storage of these mRNAs together with translation factors and other RNA-binding proteins (RBPs) in SGs ensures cell survival during stress and enables rapid SG disassembly and translation re-initiation upon recovery from stress. Importantly, impaired SG disassembly is linked to some neurodegenerative diseases, including amyotrophic lateral sclerosis and frontotemporal dementia. It is well established that in response to proteotoxic stress the UPS functions as a major PQC system by removing misfolded proteins. Accordingly, ubiquitylation is strongly induced in response to heat or oxidative stress. However, the specific subset of proteins undergoing ubiquitylation in response to distinct stimuli has remained largely elusive. Further, the contribution of ubiquitylation to stress resilience beyond the disposal of misfolded proteins is not well understood. In particular, the role of ubiquitylation in the dynamics of SG has remained controversial.^3^ In the canonical ubiquitylation pathway ubiquitin is covalently conjugated to lysine (K) residues of target proteins by an enzymatic cascade, comprised of E1-activating enzymes, E2-conjugating enzymes, and E3 ligases. Ubiquitylation can form different types of lysine-linked polymeric chains that trigger distinct downstream processes. Proteins marked with K48-chains are typically targeted to the proteasome for proteolytic degradation, whereas other chain-types mediate proteasome-independent non-proteolytic signaling functions, as exemplified by K63-linked chains that mediate the extraction of proteins from complexes. The AAA ATPase p97/VCP has an important role in both degradative and non-degradative ubiquitin signaling by extracting ubiquitylated proteins from membranes, chromatin, or protein complexes.

To better define stress-induced ubiquitylation events Maxwell et al.^2^ set out with an unbiased system-wide analysis of the cellular ubiquitylome in response to several stress stimuli: heat shock, oxidative stress (arsenite), osmotic stress (sorbitol), ultraviolet irradiation, and proteasome inhibition. Affinity enrichment of ubiquitylated proteins followed by mass-spectrometry revealed common ubiquitylation events as part of a general stress response. Importantly, however, each stress type was also associated with a distinct pattern of ubiquitylated proteins indicative of a selective role of ubiquitylation in the adaptive stress response. This is exemplified by functional annotation clustering of the heat- and arsenite-induced ubiquitylomes, which revealed common pathways, such as splicing and nucleocytoplasmic transport, but defined protein synthesis and metabolic processes as heat-specific ubiquitylation targets. Accordingly, heat- and arsenite-induced ubiquitylation exhibit a large number of alterations in ubiquitylation events that were specific for either stress stimulus. Both heat- or arsenite-induced ubiquitylation generates polyubiquitin chains consisting of different linkage-types, indicating that non-proteolytic, as well as proteolytic ubiquitin signaling, is induced. The heat shock ubiquitylome was strongly enriched for RBPs, including many constituents of SG. Accordingly, polyubiquitin chains were detected in heat-induced SGs. To explore the involvement of the ubiquitin system in the control of SG dynamics ubiquitylation was inhibited by a highly specific small-molecule inhibitor of the E1-activating enzyme. Although this did not affect SG formation, it strongly impaired the disassembly of heat-, but not arsenite-induced SGs in cancer cell lines as well as iPSC-derived neurons. Importantly, ubiquitylation was specifically required for SG clearance during the recovery phase. In the companion paper, Gwon et al.^1^ provide evidence that the SG scaffold protein G3BP1 is one key substrate for polyubiquitin-dependent disassembly of heat-induced SGs. They report that K63-linked polyubiquitylation of G3BP1 mediates its SG extraction by VCP/p97 in conjunction with the adaptor protein FAF2 ultimately resulting in granule disassembly.

The overarching concept and main conclusion from these data are that heat-induced polyubiquitylation is instrumental in preparing cells for the restart of cellular activities upon stress release. The resumption of translation, which is accompanied by the ubiquitin-dependent disassembly of SGs, is only one critical process in this recovery phase. Additional ubiquitin-mediated processes include the resumption of nucleocytoplasmic transport. Another major finding is that ubiquitylation exerts stress- and context-specific functions in SG elimination. In line with this idea, an independent study by Tolay and Buchberger found that the inhibition of ubiquitylation or VCP/p97 impaired the clearance of arsenite- and heat-induced SGs, whereas SGs induced by other stress conditions were little affected.^4^ The fact, that Tolay and Buchberger also observed involvement of ubiquitylation in the clearance of arsenite-induced SGs might be explained by cell-type-specific effects. The work by Gwon et al. also reconciles some seemingly contradictory data on the importance of autophagy in SG elimination and the role of VCP/p97 in this process. Their findings support a model where clearance of SGs upon recovery from acute stress relies on an autophagy-independent disassembly and recycling pathway, whereas autophagy-dependent elimination is important for more persistent SGs that form upon prolonged stress or are initiated by disease mutations. Importantly, Gwon et al. also revealed that mutations of VCP/p97 associated with ALS- and frontotemporal dementia impair both autophagy-dependent and independent SG clearance. Altogether, these findings therefore not only deepen our understanding of basic mechanisms in the proteotoxic stress response but also outreach to the pathogenesis of the neurodegenerative disease.

These intriguing findings also open up a series of future questions. One key question is how the ubiquitin system mediates the observed stress-specific responses. An attractive hypothesis is that stress-specific post-translational modifications drive distinct ubiquitylation events. Along this line, it has been shown that heat-induced modification with the ubiquitin-related modifier SUMO primes SG-associated proteins for ubiquitylation by the SUMO-targeted Ub ligase (StUbL) RNF4.^5^ Inhibition of the StUbL pathway impairs SG clearance pointing to an intricate interplay of ubiquitin and its cousin SUMO in stress recovery. A detailed understanding of these processes will also deepen our insight into the dynamics of disease-linked aberrant SGs.

**Fig. 1:**
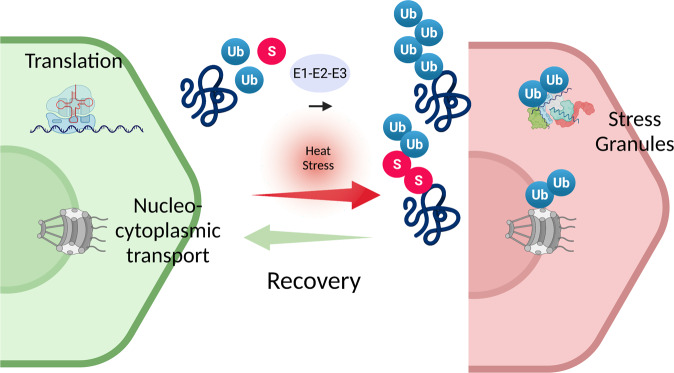
Heat stress induces polyubiquitylation of SG-associated proteins by an enzymatic E1-E2-E3 cascade. Heat-induced polyubiquitylation prepares cells for the restart of cellular activities upon stress release. The resumption of translation, which is accompanied by the disassembly of SGs is only one critical ubiquitin-regulated process in this recovery phase. Additional processes include the resumption of nucleocytoplasmic transport. The ubiquitin-related SUMO system likely contributes to heat-induced polyubiquitylation of SG-associated proteins (for details see text).
